# Inhibitory receptor CD47 binding to plasma TSP1 suppresses NK-cell IFN-γ production via activating the JAK/STAT3 pathway during HIV infection

**DOI:** 10.1186/s12967-023-04667-6

**Published:** 2023-11-30

**Authors:** Bin Lang, Meiting Wang, Zining Zhang, Yajing Fu, Xiaoxu Han, Qinghai Hu, Haibo Ding, Hong Shang, Yongjun Jiang

**Affiliations:** 1 State Key Laboratory for Diagnosis and Treatment of Infectious Diseases, NHC Key Laboratory of AIDS Immunology, National Clinical Research Center for Laboratory Medicine, The First Hospital of China Medical University, No 155, Nanjing North Street, Heping District, Shenyang, 110001 Liaoning China; 2 Key Laboratory of AIDS Immunology, Chinese Academy of Medical Sciences, Shenyang, 110001 China; 3 Key Laboratory of AIDS Immunology of Liaoning Province, Shenyang, 110001 China; 4 Collaborative Innovation Center for Diagnosis and Treatment of Infectious Diseases, 79 Qingchun Street, Hangzhou, 310003 China

**Keywords:** CD47, TSP1, IFN-γ, NK cells, JAK–STAT3, HIV infection

## Abstract

**Background:**

Natural killer (NK) cells play an important first-line role against tumour and viral infections and are regulated by inhibitory receptor expression. Among these inhibitory receptors, the expression, function, and mechanism of cluster of differentiation 47 (CD47) on NK cells during human immunodeficiency virus (HIV) infection remain unclear.

**Methods:**

Fresh peripheral blood mononuclear cells (PBMCs) were collected from people living with HIV (PLWH) and HIV negative controls (NC) subjects. Soluble ligand expression levels of CD47 were measured using ELISA. HIV viral proteins or Toll-like receptor 7/8 (TLR7/8) agonist was used to investigate the mechanisms underlying the upregulation of CD47 expression. The effect of CD47 on NK cell activation, proliferation, and function were evaluated by flow cytometry. RNA-seq was used to identify downstream pathways for CD47 and its ligand interactions. A small molecule inhibitor was used to restore the inhibition of NK cell function by CD47 signalling.

**Results:**

CD47 expression was highly upregulated on the NK cells from PLWH, which could be due to activation of the Toll-like receptor 7/8 (TLR7/8) pathway. Compared with NC subjects, PLWH subjects exhibited elevated levels of CD47 ligands, thrombospondin-1 (TSP1), and counter ligand signal regulatory protein-α (SIRPα). The TSP1–CD47 axis drives the suppression of interferon gamma (IFN-γ) production and the activation of the Janus kinase signal transducer and activator of transcription (JAK–STAT) pathway in NK cells. After treatment with a STAT3 inhibitor, the NK cells from PLWH showed significantly improved IFN-γ production.

**Conclusions:**

The current data indicate that the binding of the inhibitory receptor CD47 to plasma TSP1 suppresses NK cell IFN-γ production by activating the JAK/STAT3 pathway during HIV infection. Our results suggest that CD47 and its related signalling pathways could be targets for improving NK cell function in people living with HIV.

**Supplementary Information:**

The online version contains supplementary material available at 10.1186/s12967-023-04667-6.

## Background

The “immune inhibitory receptors” represent a heterogeneous family of transmembrane receptors that play an important role in negatively regulating immune cells [1–3]. In recent years, antibody-mediated immune inhibitory receptor blockade using antibodies against programmed death protein-1 (PD-1), also known as nivolumab, has led to great improvements in immune responses [4–6] and even in clinical outcomes of patients with neoplastic diseases [7–10]. Among these inhibitory receptors, the emergence of CD47 has attracted considerable interest.

CD47 is a transmembrane protein that is ubiquitously expressed on the surface of multiple types of cells, including tumours and diverse immune cells [11, 12]. Previous studies have reported that in oncological diseases, the interplay between CD47 overexpression in tumour cells and counter ligand signal regulatory protein-α (SIRPα) expression in macrophages initiates the “don^’^t eat me” signal, which inhibits the capacity of macrophage phagocytosis [13–16]. In addition to the surface of tumour cells, CD47 is widely expressed on immune cells. The interaction between CD47 and thrombospondin-1 (TSP1) in plasma has been shown to inhibit T-cell signalling [17, 18]. In summary, CD47 can play different functional roles in tumour or immune cells by binding to SIRPα on the cell surface or to the secreted TSP1 protein.

During HIV infection, the function of NK cells is regulated by a repertoire of inhibitory receptors. As an inhibitory receptor, the expression of CD47 on human NK cells is naturally higher than that on other lymphocytes, indicating that CD47 might be especially important for NK cell function. However, in people living with HIV, the expression level of CD47 on NK cells or whether CD47 impacts NK cell function and its associated mechanisms, especially CD47-related signalling pathways, have not been reported.

The present study is the first to explore CD47 expression and function in NK cells from people living with HIV. We also examined the expression of the CD47 ligands TSP1 and SIRPα in people living with HIV and investigated their impact on the function and mechanisms of NK cells.

## Materials and methods

### Study subjects

In this study, 50 people living with HIV were enrolled from the men who have sex with men (MSM) cohort of the First Hospital of China Medical University, and 42 HIV-negative individuals were enrolled as negative controls (NC). Demographic information and clinical characteristics of enrolled individuals are listed in Additional file 1: Table S1. The ethical review committee from the First Hospital of China Medical University approved the collection of blood samples from PLWH and written informed consent for participation in the study was obtained from all individuals.

### Determination of CD47 expression

Fresh peripheral blood mononuclear cells (PBMCs) were collected from people living with HIV and NC subjects. CD4^+^ T cells, CD8^+^ T cells and total NK cells were defined as CD3^+^CD4^+^, CD3^+^CD8^+^, and CD3^−^CD56^+^, and the three NK cell subsets were identified as CD3^−^CD56^bri^CD16^+/−^, CD3^−^CD56^dim^CD16^+^, and CD3^−^CD56^−^CD16^+^. All samples were collected using an LSR II cytometer (BD Biosciences), and the data were analysed using FlowJo V10.0 software (BD Biosciences).

### Measurement of plasma TSP1 levels

Plasma TSP1 levels were measured using an enzyme-linked immunosorbent assay (ELISA). Peripheral whole blood (2–3 mL) was collected from PLWH and NC subjects in ethylenediaminetetraacetic acid (EDTA) vacuum blood collection vessels (BD Biosciences). The level of TSP1 in plasma was measured by the Human Thrombospondin-1 Quantikine ELISA Kit (R&D), and Curve Expert (Hyams Development) was used to construct the ELISA standard curve for analysis.

### Expression of CD47 on CD4^+^ and CD8^+^ T cells and NK cells stimulated by HIV proteins Gag and Env and a TLR7/8 agonist

EDTA-anticoagulated whole blood from NC subjects was collected, and PBMCs were extracted. In the control group, 1 μL phosphate-buffered saline (PBS) was added per well. The treatment group was divided into the Gag (Prospec) treatment group (0.5, 1, and 2 μg/mL), Env (Prospec) treatment group (0.5, 1, and 2 μg/mL), Gag (2 μg/mL) + Env (2 μg/mL) treatment group, and TLR7/8 agonist-R848 (Med Chem Express) treatment group (2 μg/mL). Flow cytometry surface staining was described above and analysed using an LSR II cytometer.

### Effect of the TSP1–CD47 axis on NK cell activation, proliferation, and function (CD69, Ki-67, and IFN-γ)

For the determination of CD69 and IFN-γ levels in NK cells, cells were processed as described above, 1 μL PBS was added to each well in the control group, and TSP1 recombinant protein (R&D, 1000 ng/mL) was added to each well in the treatment group. Then, 5 μL PE-CD69 (BioLegend) flow fluorescent antibody and 5 μL allophycocyanin (APC)-conjugated IFN-γ (BioLegend) fluorescent antibodies were added to the cells. The Fix/Perm Buffer Set (BioLegend) and BV421-Ki-67 (BD) antibody were used to detect Ki67 expression in NK cells. One microlitre of PBS was added to each well in the control group, and TSP1 recombinant protein (250, 500, and 1000 ng/mL) was added to each well in the treatment group. IFN-γ production of NK cells was stimulated by IL-12 (10 ng/mL) (R&D) and IL-15 (50 ng/mL) (R&D). After incubation at 37 °C for 24 h, a FACS Canto II cytometer was used to detect relevant markers.

### Determining the phosphorylation levels of the NFAT and STAT3 in NK cells

Purified NK cells were isolated by an EasySep™ Human NK Cell Isolation Kit (Stem Cell) from NC subjects. NK cells were precultured with 2 μg/mL anti-SIRPα (Santa Cruz) or 1000 ng/mL recombinant TSP1 (Santa Cruz) at 37 °C for 1 h. After the treatment, NK cells were stimulated by IL-12 (10 ng/mL) and IL-15 (50 ng/mL) for 15 min. Then, 5 μL AF488-anti-NFAT-Phospho (CST) or 5 μL PE-STAT3-Phospho (BioLegend) fluorescent antibody was added to the tubes. The tubes were incubated in the dark at 4 °C for 20 min. The phosphorylation levels of NFAT and STAT3 in NK cells were determined by flow cytometry using a BD Canto II instrument.

### RNA-Seq analysis

A total of three samples of purified NK cells were collected from NC subjects. In the control group, NK cells were treated with 2 μL PBS for 1 h. In the treatment group, NK cells were treated with 1000 ng/mL recombinant TSP1 for 1 h. Transcriptome sequencing was performed using the BGISEQ-500 platform by Shenzhen Huada Gene Technology Co., Ltd. Transcriptome results were analysed using the Dr. Tom platform (biosys.bgi.com, Huada gene). The filtering software SOAPnuke (https://github.com/BGI-flexlab/SOAPnuke) was independently developed by Huada for filtering. The GRCh38.p13 reference genome from NCBI was used as the reference.

### Statistical analysis

A flow chart was constructed with FlowJo V10.0 software (BD Biosciences). The experimental results were plotted and statistically analysed by GraphPad Prism 8 (GraphPad Software) and SPSS 24.0 software (IBM). For two independent samples, the nonparametric Mann–Whitney U test was used to compare the differences in quantitative data between the two groups. For paired data, the paired t test or Wilcoxon paired signed-rank test was applied. *P* < 0.05 was considered statistically significant.

## Results

### CD47 expression is upregulated on the NK cells of people living with HIV infection.

**Fig. 1:**
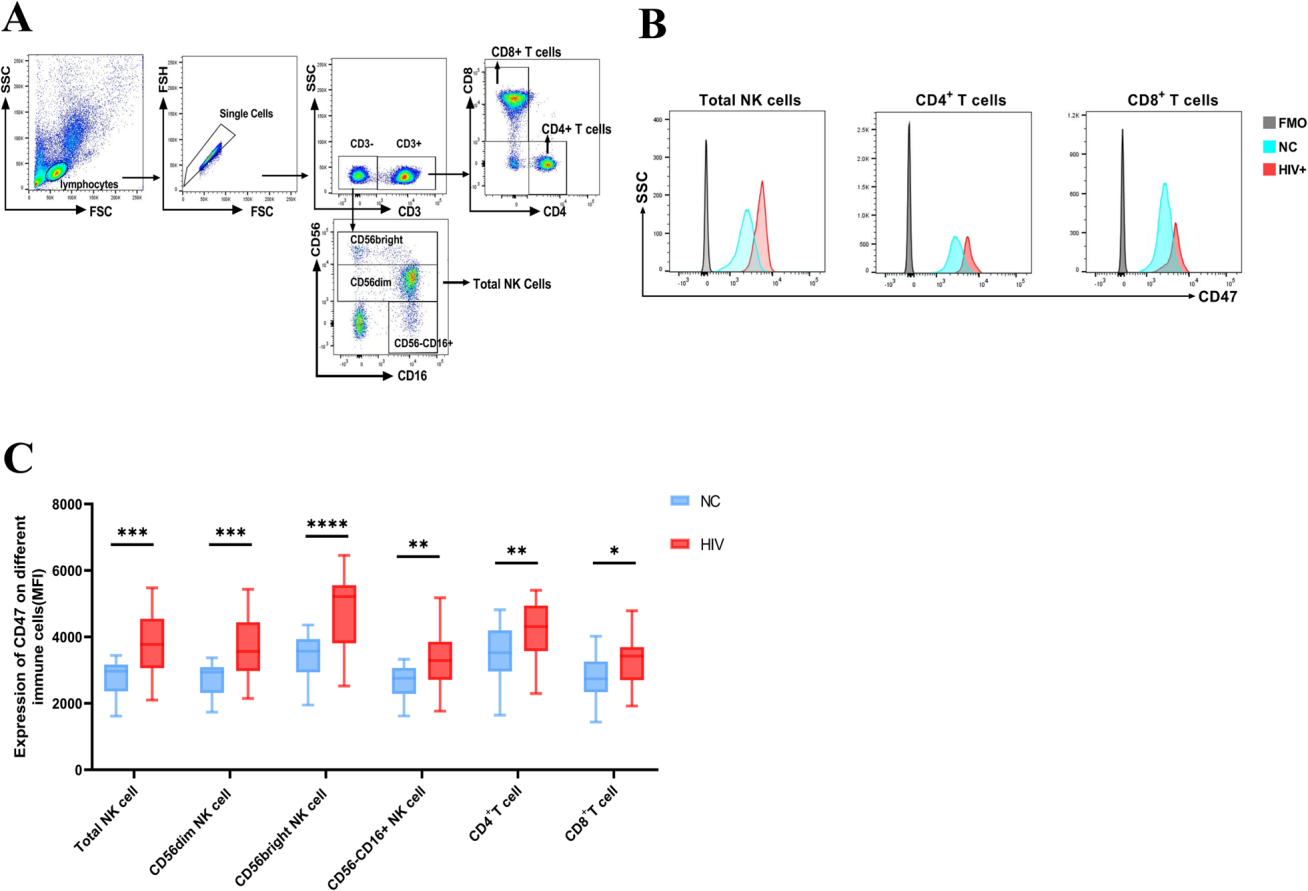
CD47 expression on NK cells and CD4^+^ and CD8^+^ T cells is upregulated in PLWH. **A** Gating strategy for human total natural killer (NK) cells and NK cell subsets and CD4^+^ and CD8^+^ T cells. Total NK cells were identified from CD3^−^ lymphocytes by their expression of CD16 and/or CD56. Three NK cell subsets were gated: (1) CD56^dim^ NK cells (CD3^−^CD56^dim^CD16^−/+^), (2) CD56^bright^ NK cells (CD3^−^CD56^bri^CD16^−/+^) and (3) CD56^−^CD16^+^ NK cells. CD4^+^ and CD8^+^ T cells were identified from lymphocytes using a CD3^+^CD4^+^ gate or CD3^+^CD8^+^ gate. **B** Gating strategy and representative cytometry dot plots of CD47 expression in total NK cells and CD4^+^ and CD8^+^ T cells in people with HIV and NC subjects. Grey, fluorescence minus one (FMO); blue, HIV-negative controls; red, people living with HIV. **C** Mean fluorescence intensity (MFI) of CD47 expression on total NK cells, CD56^dim^ NK cells, CD56^bright^ NK cells, CD56^−^CD16^+^ NK cells, CD4^+^ T cells and CD8^+^ T cells in people with HIV (n = 28) and NC subjects (n = 19). A nonparametric Mann–Whitney U test was used to compare groups, and error bars represent median and interquartile range; *p < 0.05, **p < 0.01, ***p < 0.001, ****p < 0.0001

To assess the alteration of CD47 expression in people living with HIV, we conducted measurements on NK cells, CD4^+^ T cells, and CD8^+^ T cells from human PBMCs by flow cytometry (gating strategy and representative plots are presented in Fig. 1A, B). We found that the expression of CD47 mean fluorescence intensity (MFI) was significantly higher on total NK cells and three NK subsets (CD56^dim^ NK, CD56^bright^ NK, and CD56^−^CD16^+^ NK cells) in the PLWH group than in the NC group (Fig. 1C). Similarly, we also observed significantly upregulated CD47 expression on the CD4^+^ T and CD8^+^ T cells from the PLWH group compared to the NC group. Overall, these findings indicate that the expression of CD47 is upregulated on NK cells during HIV infection.

### CD47 expression can be upregulated on NK cells by HIV proteins, Gag and Env, or TLR7/8 activation

**Fig. 2:**
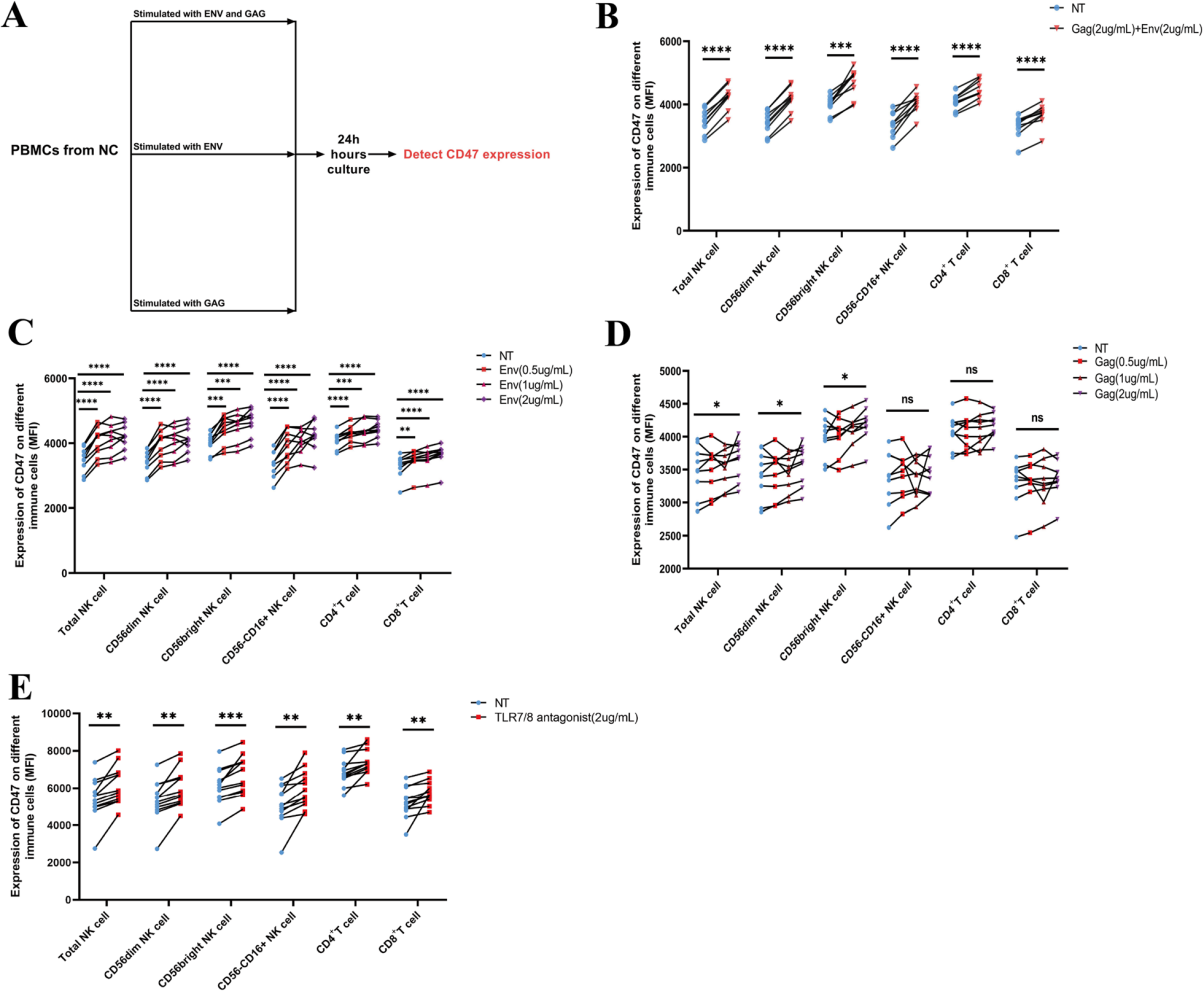
CD47 expression can be upregulated in NK cells by HIV proteins or TLR7/8 activation. **A** Schematic diagram of experiments using PBMCs from the NC group treated with different concentrations (0, 0.5, 1, and 2 μg/mL) of the HIV-1 proteins Gag and Env for 24 h as single treatments and in combination. **B** MFI of CD47 expression in human peripheral blood mononuclear cells (PBMCs) total NK cells, CD56^dim^ NK cells, CD56^bright^ NK cells, CD56^−^CD16^+^ NK cells and CD4^+^ and CD8^+^ T cells from the control (NC) group (n = 9) after 24 h of stimulation with Gag (2 μg/mL) and Env (2 μg/mL) or with no stimulation. **C**, **D** MFI of CD47 expression in human PBMC total NK cells, CD56^dim^ NK cells, CD56^bright^ NK cells, CD56^−^CD16^+^ NK cells, CD4^+^ T cells and CD8^+^ T cells from the NC group (n = 9) after 24 h of stimulation with Gag (0.5, 1, and 2 μg/mL) or Env (0.5, 1, and 2 μg/mL) or with no stimulation. **E** MFI of CD47 expression on human PBMC total NK cells, CD56^dim^ NK cells, CD56^bright^ NK cells, CD56^−^CD16^+^ NK cells, CD4^+^ T cells, and CD8^+^ T cells from the NC group (n = 12) after 24 h of stimulation with the TLR7/8 agonist R848 (2 μg/mL) or with no stimulation. Paired t- or Wilcoxon signed-rank tests were used for paired-group comparisons, and error bars represent the median and interquartile range; *p < 0.05, **p < 0.01, ***p < 0.001, ****p < 0.0001; *ns* no significance, *NT* no treatment

Although we observed a significant upregulation of CD47 on NK cells during HIV infection, the mechanism of CD47 upregulation during HIV infection remains to be confirmed. Initially, our speculation revolved around the possibility that HIV proteins released during infection may trigger the upregulation of CD47 expression on NK cells. Therefore, we first stimulated PBMCs from the NC group with different concentrations (0, 0.5, 1, and 2 μg/mL) of HIV-1 proteins, Gag and Env, as single treatments and in combination (Fig. 2A). At 24 h poststimulation, we found that the combination Gag (2 μg/mL) and Env (2 μg/mL) treatment group, compared to the no-treatment group, exhibited significantly upregulated CD47 expression on total NK cells, CD4^+^ T cells and CD8^+^ T cells (Fig. 2B). Furthermore, higher concentrations of Env dose-dependently upregulated CD47 expression on NK cells, CD4^+^ T cells and CD8^+^ T cells (Fig. 2C); however, a concentration of 2 μg/mL Gag treatment only upregulated CD47 expression on total NK cells, CD56^dim^ NK cells, and CD56^bright^ NK cells but did not affect CD47 expression on CD56^−^CD16^+^ NK cells, CD4^+^ T cells, or CD8^+^ T cells (Fig. 2D).

Furthermore, Tal et al. [19] reported that the activation of Toll-like receptor 7/8 (TLR7/8) led to the upregulation of CD47 on human dendritic cells (DCs) and monocytes in vitro. This finding suggests that, apart from stimulation by HIV protein antigens, the release of HIV single-stranded RNA (ssRNA) may also contribute to the upregulation of CD47 on NK cells during HIV infection. Hence, we specifically used a TLR7/8 agonist (resiquimod, R848) to activate TLR7/8 and observe the expression of CD47 on NK cells. After treatment with R848 (2 μg/mL) for 24 h, CD47 expression on NK cells from the NC group was significantly upregulated compared with that on NK cells from the nontreatment group (Fig. 2E). Altogether, the upregulation of CD47 on NK cells during HIV infection can be attributed to the stimulation caused by HIV proteins or HIV ssRNA.

### The levels of the CD47 ligands TSP1 and SIRPα are elevated during HIV infection

**Fig. 3:**
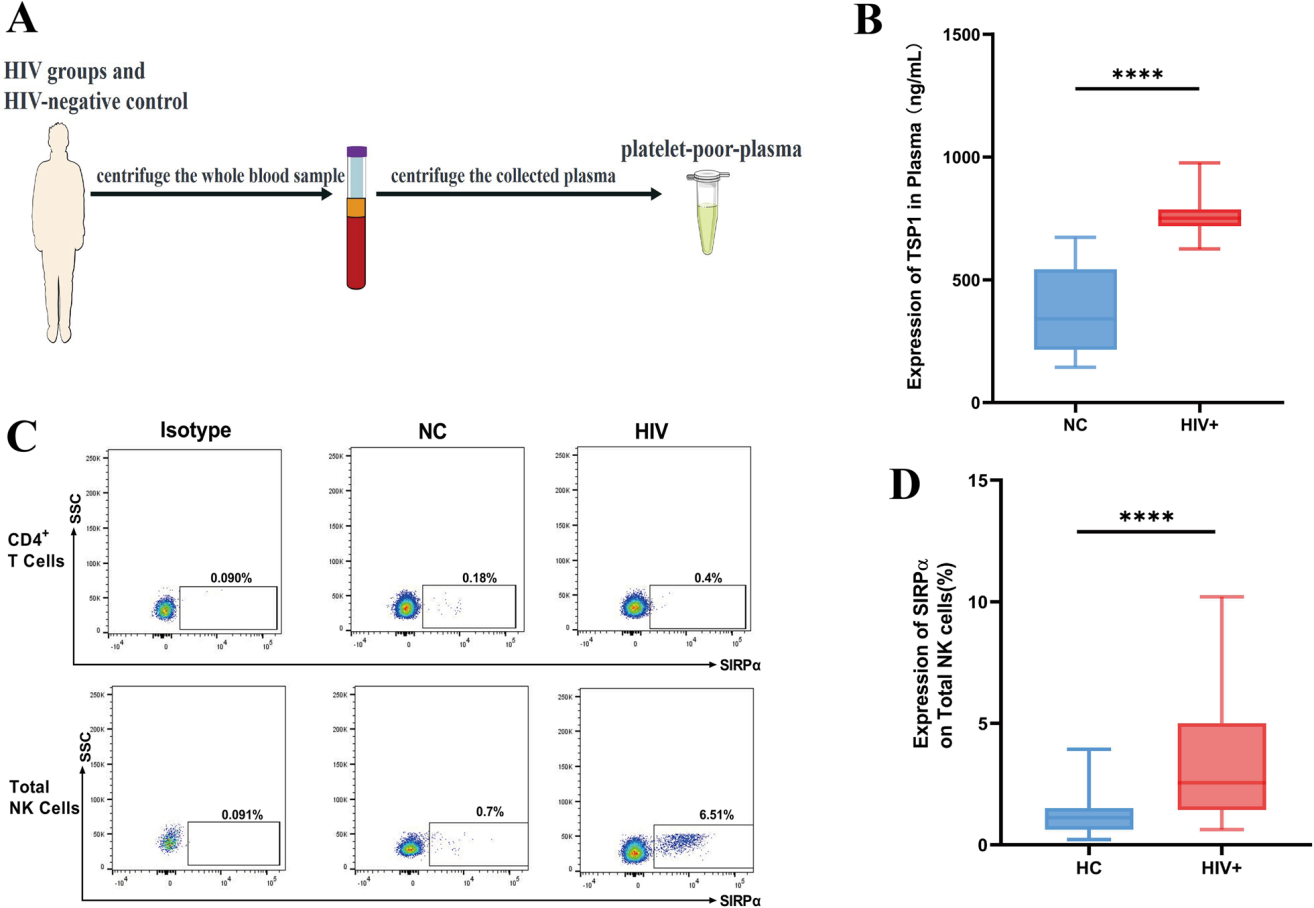
The expression of the CD47 ligands TSP1 and SIRP is significantly upregulated in PLWH. **A** Schematic diagram of experiments using poor-platelet plasma from the PLWH and NC groups to measure thrombospondin (TSP1) expression levels using an enzyme-linked immunosorbent assay (ELISA). **B** TSP1 expression level in poor-platelet plasma from the PLWH group (n = 22) and NC group (n = 23). **C** Gating strategy and representative cytometry dot plots for signal regulatory protein alpha (SIRPα) expression in human total NK cells and CD4^+^ T cells. SIRPα expression was gated based on an isotype control. **D** Percentages of SIRPα expression in total NK cells from people with HIV (n = 28) and NC subjects (n = 27). A nonparametric Mann–Whitney U test was used to compare groups, and error bars represent median and interquartile range; ****p < 0.0001

The plasma expression levels of TSP1, a ligand of CD47, in PLWH remain undetermined. Therefore, we investigated TSP1 expression levels in the plasma from PLWH and NC using ELISA (Fig. 3A). Our experiment indicated that the expression levels of TSP1 were higher in the PLWH group than in the NC group (Fig. 3B).

SIRPα is another known ligand of CD47 that is mainly expressed on dendritic cells or phagocytes [12]. However, SIRPα expression on CD4^+^ T cells during HIV infection has not been previously reported. As CD4^+^ T cells are target cells for HIV, we first examined the expression of SIRPa on CD4^+^ T cells and found that the expression level of SIRPα on the CD4^+^ T cells from the NC group was extremely low based on the flow cytometry results (Fig. 3C), indicating that the increased CD47 levels on the surface of NK cells may not primarily interact with SIRPα on CD4^+^ T cells. Additionally, Deuse et al. [20] reported that SIRPα could serve as a potent inhibitory receptor on NK cells by binding to CD47 and transmitting CD47-induced inhibitory signals to NK cells. Thus, we further investigated the expression of SIRPα on NK cells during HIV infection. Our experiment showed that the expression levels of SIRPα on total NK cells were higher in the PLWH group than in the NC group (Fig. 3D). Based on the above results, our findings suggest that both the expression of the soluble ligand TSP1 in plasma and SIRPα on NK cells are elevated during HIV infection.

### The TSP1–CD47 axis acts as an inhibitory signal for NK cell functions

**Fig. 4:**
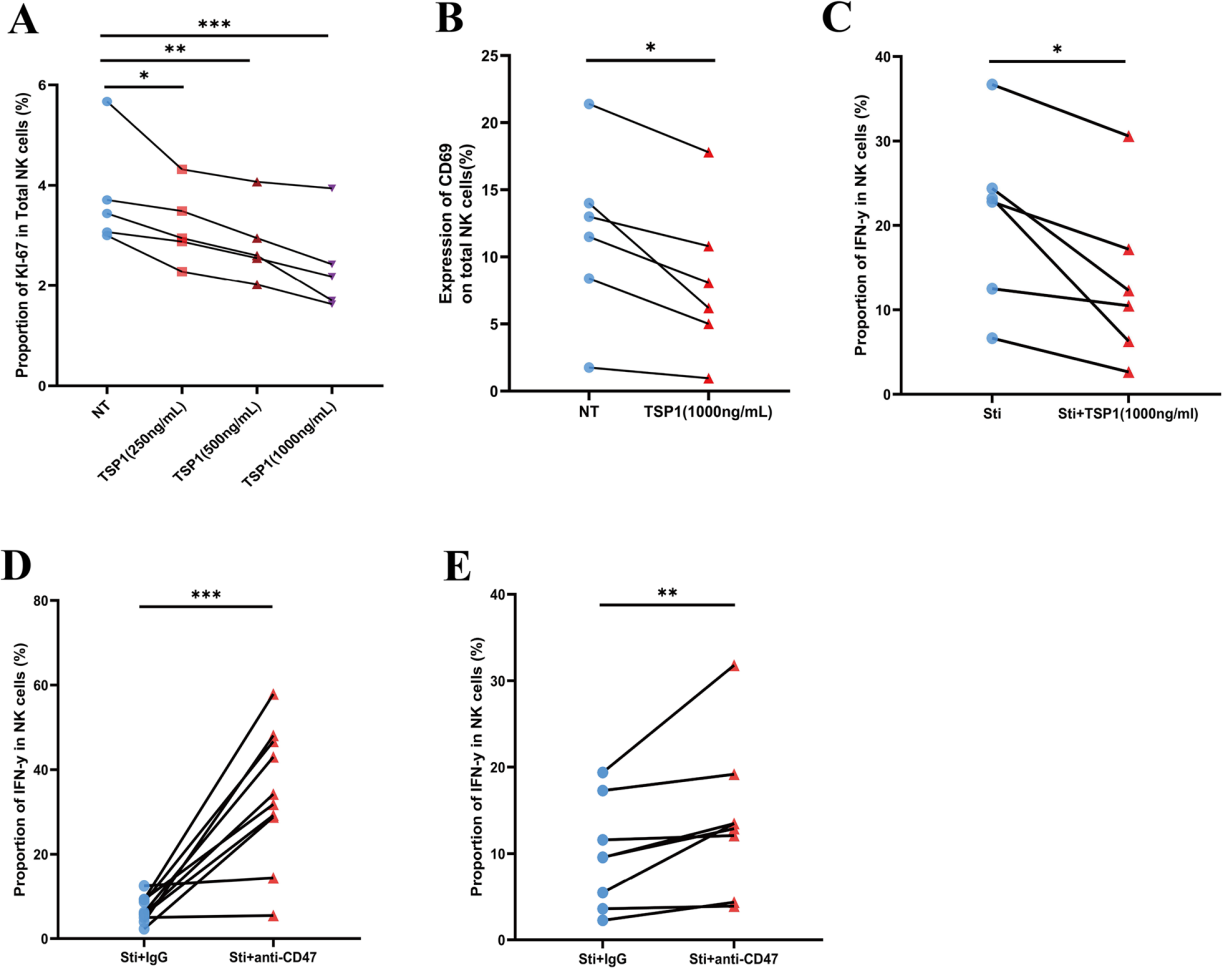
The TSP1–CD47 axis inhibits NK cell activation, proliferation, and IFN-γ production. **A** Paired comparisons of the percentage of Ki-67 expression in total NK cells with different concentrations of recombinant TSP1 (0, 250, 500, and 1000 ng/mL) treatment (n = 5). **B** Paired comparisons of the percentage of CD69 expression in total NK cells with or without recombinant TSP1 (1000 ng/mL) treatment (n = 6). **C** Paired comparisons of the percentage of interferon gamma (IFN-γ) expression in total NK cells with or without recombinant TSP1 (1000 ng/mL) treatment (n = 6). **D** Paired comparisons of the percentage of IFN-γ expression in total NK cells with anti-CD47 antibody (2 μg/mL) or IgG-control (2 μg/mL) treatment (n = 10). **E** Paired comparisons of the percentage of IFN-γ expression in total NK cells from PLWH with Anti-CD47 antibody (2 μg/mL) or IgG-control (2 μg/mL) treatment (n = 7). Paired t- or Wilcoxon signed-rank tests were used to make paired-group comparisons, and error bars represent median and interquartile range; *p < 0.05, **p < 0.01, ***p < 0.001; *ns* no significance, *NT* no treatment

To determine whether the TSP1–CD47 interaction inhibits the proliferation and activation of primary human NK cells, we used recombinant TSP1 protein cocultured with NK cells (Additional file 1: Fig. S1A) and found that compared to the no treatment (NT) group, the TSP1 treatment group demonstrated a dose-dependent reduction in the levels of the NK cell proliferation marker Ki-67 after 24 h (Fig. 4A). Furthermore, we used recombinant TSP1 protein to treat NK cells for 24 h and found that CD69 expression, as an assessment of NK cell activation, was significantly decreased compared to that in the NT group (Fig. 4B).

Next, we assessed the effect of the TSP1–CD47 axis on the function of NK cells (Additional file 1: Fig. S1B). We found that after culturing with IL-12 and IL-15 for 24 h, NK cells showed significant inhibition of IFN-γ production in the presence of TSP1 (Fig. 4C).

We further explored whether IFN-γ production by NK cells can be restored by CD47 blockade. Compared to the IgG control, CD47 blocking treatment restored IFN-γ production in NK cells from the HIV-negative control group (Fig. 4D). Additionally, we further blocked CD47 on the NK cells from people living with HIV by using an anti-CD47 antibody. Consistent with the results of CD47 blockade in the HIV-negative control group, IFN-γ production was significantly restored in NK cells compared to NK cells treated with IgG (Fig. 4E). Moreover, we investigated whether TSP1 requires CD47 expression to play an inhibitory role in NK cells. Our data show that CD47 blockade led to an enhancement of IFN-γ production by NK cells in the presence of TSP1 when compared with the IgG control (Additional file 1: Fig. S1C). Altogether, our results indicate that CD47 acts as a strong inhibitory receptor with or without TSP1 in NK cells.

### Blockade of SIRPα can restore NK cell IFN-γ production by activating the NFAT pathway

SIRPα has been reported to exist on the surface of NK cells as an inhibitory receptor that binds to CD47 [20]. Therefore, we explored the effects of SIRPα on IFN-γ production in NK cells from PLWH (Additional file 1: Fig. S2A). An anti-SIRPα antibody was used to block SIRPα, and we found for the first time that IFN-γ production by NK cells was significantly enhanced (Additional file 1: Fig. S2B). Our previous study showed that NFAT signalling pathways play an important role in NK cell IFN-γ production [21]. Thus, we investigated whether SIRPα blockade could impact NFAT signalling pathways (Additional file 1: Fig. S2C). After treatment with SIRPα blocking antibody (2 μg/mL) for 1 h, the levels of NFAT activity in NK cells were increased compared to those in NK cells treated with the IgG control (Additional file 1: Fig. S2D). Overall, these results indicate that blockade of SIRPα could restore NK cell IFN-γ production by activating the NFAT pathway.

### RNA-seq reveals that the TSP1–CD47 axis drives the activation of the JAK–STAT3 pathway in NK cells

**Fig. 5:**
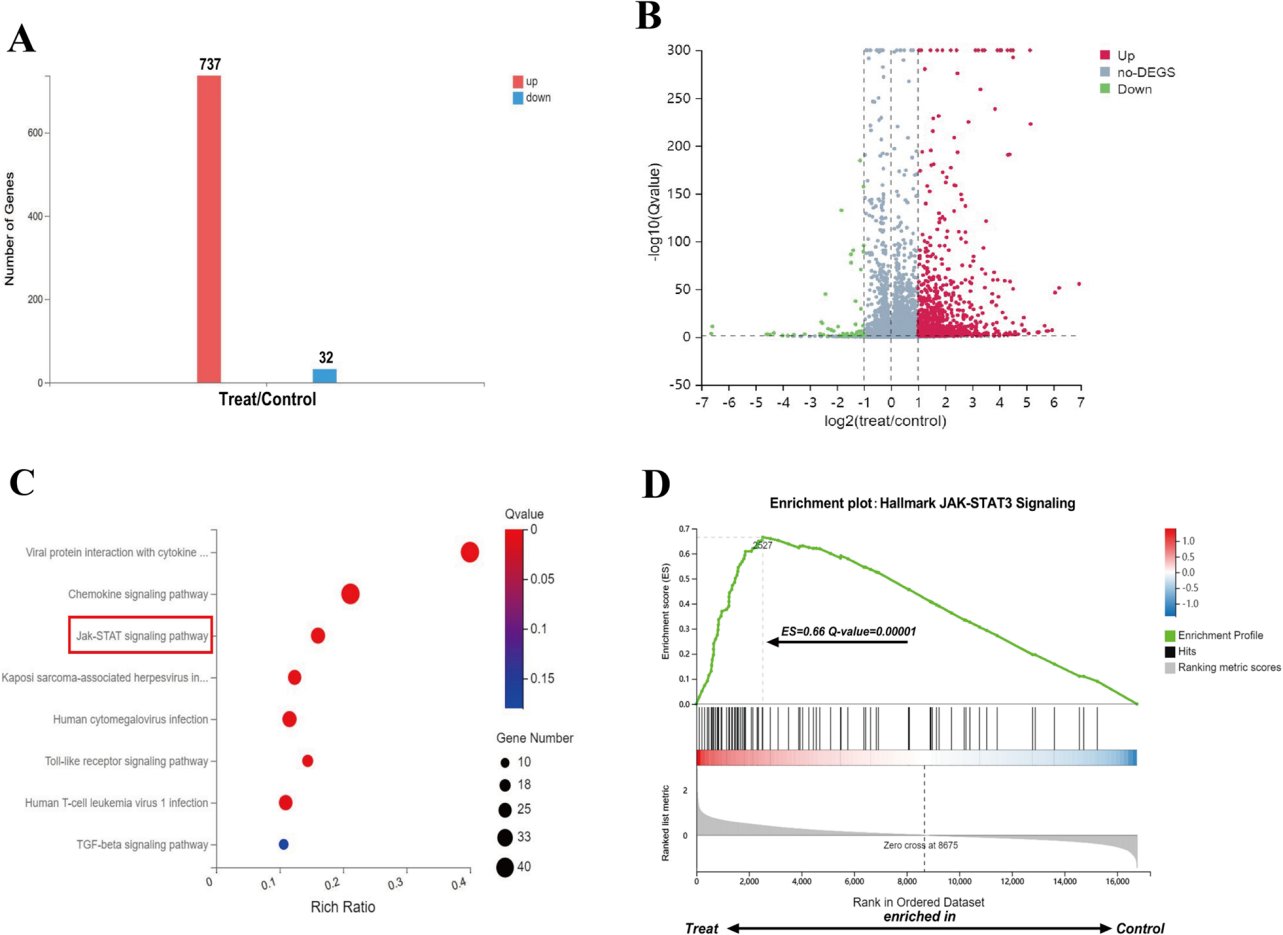
Transcriptome analysis of the effect of the TSP1–CD47 axis on the NK cell pathway. **A** Histogram of differentially expressed gene (DEG) expression of NK cells from NC in the control group and TSP1-treatment group. Red bar is the gene upregulated in TSP1-treated NK cells, and blue bar is the gene downregulated in TSP1-treated NK cells (n = 3, log_2_|FC| > 1, Q-value < 0.001). **B** Volcano plot of differentially expressed genes (DEGs) in NK cells from NC in the Control group and TSP1-treatment group. The horizontal axis represents the FC after the log_2_ conversion, and the vertical axis represents the Q-value after the log_2_ conversion (n = 3). **C** Gene Ontology (GO) enrichment analysis of differentially expressed genes (DEG) in NK cells from the control group and TSP1 treatment group. The bubble colour represents the significance of enrichment (Q-value), the bubble size represents the number of enriched differentially expressed genes, the horizontal axis is the enrichment ratio, and the vertical axis is the enrichment pathway category (Rich Ratio = Term Candidate Gene Num/Term Gene Num). **D** Gene set enrichment analysis (GSEA) of NK cell differentially expressed gene (DEG) enrichment pathways in the control group and TSP1 treatment group. A positive enrichment score (ES) indicates enrichment in NK cells of the TSP1-treatment group, while a negative value indicates enrichment in NK cells of the control group, and the Q-value is the P value corrected by the false discovery rate (FDR). The vertical axis represents the enrichment score, and the horizontal axis black vertical line represents pathway-related genes

To further explore the potential signalling pathway by which the TSP1–CD47 axis affects NK cells, we pretreated NK cells from NC subjects with or without TSP1 and conducted RNA-seq. The transcriptome results showed that compared to the control group, 737 genes were upregulated and 32 genes were downregulated in the TSP1 treatment group (threshold: log2|FC| > 1, Q-value < 0.001), as shown in Fig. 5A, B. Through a Gene Ontology (GO) enrichment analysis of upregulated genes in the TSP1 treatment group, we found for the first time that the JAK–STAT3 signalling pathway was highly enriched (Fig. 5C). Further gene set enrichment analysis (GSEA) also revealed that specific factors of the JAK–STAT3 pathway were enriched in the upregulated genes of the TSP1 treatment group (Fig. 5D). Since STAT3 contributes to the inhibition of NK cell function, we speculated that the TSP1–CD47 axis negatively regulates NK cell function by activating the STAT3 signalling pathway.

### Blocking STAT3 phosphorylation restores IFN-γ production in NK cells

**Fig. 6:**
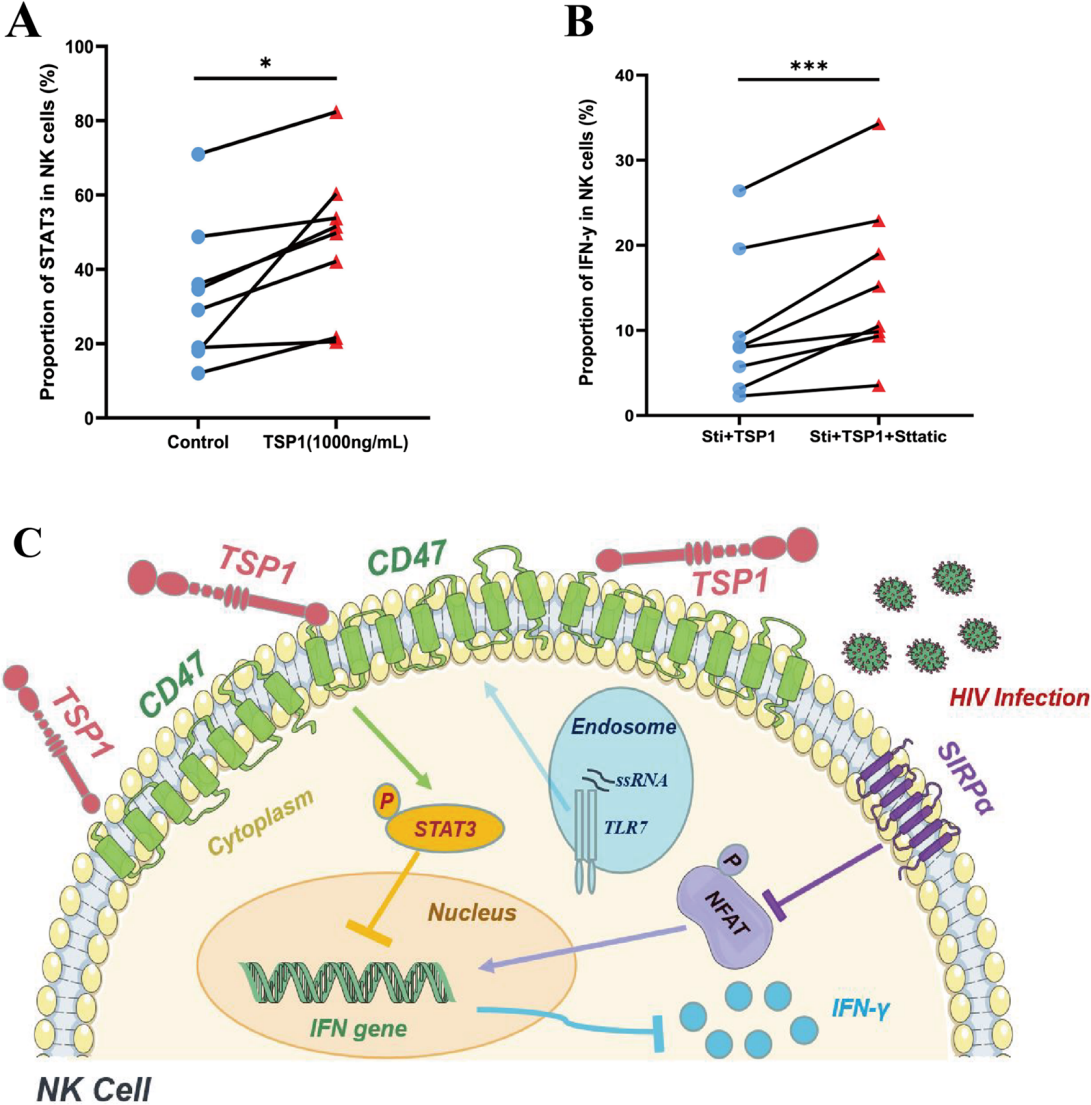
The TSP1–CD47 axis activates the STAT3 signalling pathway in NK cells. **A** Paired comparisons of the percentage of NFAT phosphorylation in total NK cells from the NC group with or without recombinant TSP1 (1000 ng/mL) treatment (n = 8). **B** Paired comparisons of the percentage of IFN-γ expression in total NK cells from PLWH with recombinant TSP1 (1000 ng/mL) and with or without STAT3-inhibitor Stattic (2 µmol/L) treatment (n = 8). A paired t test was used for paired-group comparisons, and error bars represent the median and interquartile range; *p < 0.05, ***p < 0.001. **C** Model of CD47-related signalling influencing NK cell function in HIV infection. During HIV infection, the TLR7/8 signalling pathway recognizes pathogen-associated molecular patterns and mediates the upregulation of CD47 expression in NK cells. Moreover, CD47 binds to its soluble ligand TSP1 in plasma, activates phosphorylation of the STAT3 signalling pathway, and inhibits IFN-γ production in NK cells. The expression of another CD47 ligand, SIRPα, was upregulated on NK cells, further limiting IFN-γ production by inhibiting NFAT signal pathway phosphorylation and causing NK cells to fail to exert antiviral effects in HIV infection

To validate the RNA-seq results, flow cytometry was used to identify whether the TSP1–CD47 axis activated the JAK–STAT3 signalling pathway in NK cells (Additional file 1: Fig. S3A). As expected, compared to the control treatment, TSP1 treatment significantly increased STAT3 phosphorylation in NK cells (Fig. 6A). To determine the effects of the TSP1–CD47 axis mediation of STAT3 activation on NK function, we treated NK cells from people with HIV with a STAT3 inhibitor, Stattic (Additional file 1: Fig. S3B). As expected, in the presence of TSP1, IFN-γ production in Stattic-treated NK cells was significantly restored compared with that in control-treated NK cells (Fig. 6B**)**. Overall, we can confirm that the TSP1–CD47 axis in HIV infection leads to the phosphorylation of STAT3, which inhibits IFN-γ production by NK cells.

## Discussion

As the first line of innate immune defence, NK cells play a vital role in the process of immune clearance and surveillance [22, 23]. However, the upregulated expression of inhibitory receptors can negatively affect NK cell function. Among the various inhibitory receptors, CD47 was recently reported to be highly expressed in various types of cancers, resulting in NK cell dysfunction [14, 24, 25]. Nevertheless, the expression, function, and mechanism of CD47 in relation to NK cells during HIV infection are still unclear.

In this study, we found that CD47 expression was significantly upregulated in people living with HIV and that this upregulation was substantially higher on NK cells than on both CD4^+^ and CD8^+^ T cells. Importantly, we observed that the expression level of CD47 was higher in all of the people living with HIV than that in the HIV-negative controls despite receiving HAART treatment. This phenomenon might be because chronic HIV infection can cause paralysis of NK cell immune function [26], which then induces the upregulation of CD47. In addition, HIV may evade the immune system's clearance of infected cells by inducing an increase in CD47 expression levels on the cell surface, thereby emitting a “do not eat me” signal to the immune system. Overall, CD47 expression levels remained elevated after HAART treatment, so we further investigated the potential factors leading to the upregulation of CD47.

Our results demonstrated that CD47 exhibits dose-dependent upregulation on NK cells and CD4^+^ and CD8^+^ T cells after in vitro stimulation with HIV Env recombinant proteins. However, the precise mechanism by which HIV-1 Env mediates the upregulation of CD47 remains unclear. The HIV-1 Env recombinant protein might be recognized by antigen-presenting cells and then processed into peptides presenting through MHC molecules [27]. HIV-1 Env peptides could induce the release of proinflammatory cytokines in PBMCs, including tumour necrosis factor-alpha (TNF-α), interleukin-6 (IL-6) and others [28–30]. These inflammatory factors can lead to overactivation of the immune cells and serve as a negative feedback loop, and CD47 was upregulated on NK cells, CD4^+^ and CD8^+^ T cells. Moreover, proinflammatory cytokines such as IFN-α and TNF-α have been reported to directly upregulate CD47 expression [31, 32]. Although HIV-1 Env/Gag proteins have been commonly used to conduct experiments to observe the effect of HIV infection on lymphocyte function and receptor expression in many studies [28, 33, 34], the concentration of purified HIV proteins is unlikely to be physiological. Thus, the upregulation of CD47 on NK cells during HIV infection should be further validated by inactivated HIV or HIV-derived virus-like particles in future studies.

Furthermore, Tal et al. [19] also reported that multiple pathogen agents in both human and mouse cells could cause CD47 upregulation, indicating that CD47 upregulation could occur in response to host recognition of pathogens via pattern recognition receptor (PRR) stimulation. Via the TLR7/8 stimulation agonist R848, a marked induction of upregulation of CD47 on NK cells and CD4^+^ and CD8^+^ T cells was observed, showing that TLR7/8 activation or HIV viral proteins can lead to a strong increase in CD47 expression levels during HIV infection. Notably, as an emerging strategy in HIV vaccine development, adjuvanting HIV Env with a TLR7/8 agonist has been shown to induce rapid immune cell infiltration and enhance HIV envelope antibody responses [35, 36]. Moreover, TLR7/8 activation was also reported to limit the size of the HIV reservoir and delay viral rebound after discontinuation of ART [37–39]. However, we found that the activation of the TLR7/8 signalling pathway could induce the upregulated expression of CD47, which seems to be the opposite of the positive control of HIV infection by the TLR7/8 signalling pathway. We believe that, similar to coinhibitory checkpoints such as PD-1, the upregulation of CD47 expression can be regarded as a negative feedback regulatory process to prevent overactivation of the inflammatory response. In addition, it is crucial to note that tissue localization may differently modulate the development and function of human NK cells. The distribution and functionality of NK cells vary in different tissues [40–42]. Most NK cells are located in blood and highly vascularized tissues, such as bone marrow, spleen, and lungs. Immature NK cells primarily exist in lymph nodes, tonsils, and the gastrointestinal tract. Nonetheless, there is a lack of studies exploring the expression of CD47 on NK cells residing in various tissues or organs. Therefore, the impact of CD47 on NK cells originating from different tissues during HIV infection warrants a more thorough investigation.

Given that CD47 is highly expressed on NK cells, the expression of CD47-related ligands during HIV infection is very important. We found that the levels of CD47 soluble receptor TSP1 are elevated in the plasma of people living with HIV. Previous investigations on SIRPα, another ligand of CD47, have mainly focused on macrophages [13, 16, 39, 43], and whether CD4^+^ T cells express SIRPα has not yet been reported. We first found that SIRPα was mostly not expressed on the surface of CD4^+^ T cells in both HIV and HIV-negative individuals, indicating that CD47 on NK cells and SIRPα on CD4^+^ T cells may not play a specific role. Recently, Deuse et al. [20] demonstrated that SIRPα can also be expressed as an inhibitory receptor on NK cells from mouse and healthy donors. Thus, we examined the expression of SIRPα on NK cells and found that the expression of SIRPα on NK cells in people living with HIV was significantly upregulated, indicating that SIRPα is likely to affect the function of NK cells during HIV infection. Our main focus was to examine the expression of SIRPα as a CD47 ligand on HIV target CD4^+^ T cells and its role as an inhibitory receptor on NK cells. However, analysing the expression of SIRPα on dendritic cells and macrophages would contribute to a better understanding of the interaction between CD47 and SIRPα during HIV infection. The interaction between CD47 on NK cells and SIRPa on dendritic cells or phagocytes during HIV infection, which is a limitation of this study, is worthy of further research in the future.

By using a SIRPα blocking antibody, we found that the IFN-γ production of NK cells from people living with HIV was restored. In addition, our team previously demonstrated that the NFAT signalling pathway is important for IFN-γ production in NK cells [21]. Thus, we next determined the impact of SIRPα on the NFAT signalling pathway and first found that SIRPα blockade led to a significant increase in the phosphorylation level of NFAT in NK cells, indicating that SIRPα regulates the production of IFN-γ in NK cells by affecting the NFAT signalling pathway.

In addition to SIRPα, we also determined the effect of TSP1 on NK cells. We found that the TSP1–CD47 axis significantly reduced the expression levels of CD69 and Ki-67, the activation and proliferation indicators of human NK cells. In addition, the TSP1–CD47 axis also significantly suppressed IFN-γ production in NK cells. However, the mechanism of the TSP1–CD47 axis and its downstream pathway in NK cells has not been reported, so we further explored the related mechanism.

Through RNA-seq analysis and flow cytometry verification, we found for the first time that the TSP1–CD47 axis led to significant activation of the JAK–STAT3 signalling pathway in NK cells. Under normal physiological conditions, STAT3 can be regarded as an important factor for regulating the balance between cell proliferation and apoptosis and participating in the process of antigen tolerance and antigen presentation [44]. Previous studies have demonstrated that STAT3 plays a key role in the regulation of NK cell function [45–47]. Gotthardt et al. [48] knocked out the STAT3 gene in mouse NK cells and found that the growth and proliferation of NK cells were normal but showed a stronger killing capability towards tumour cells. By using a STAT3 inhibitor, we also successfully reversed the inhibition of NK cell IFN-γ production, confirming that the TSP1–CD47 axis does indeed regulate the JAK–STAT3 pathway.

## Conclusions

Altogether, our current study and previous studies indicate that CD47 blockade has great potential applicability for HIV treatment. During HIV infection, the expression of CD47 is significantly upregulated in people living with HIV, and TLR7/8 signalling leads to upregulation of the expression of CD47 on NK cells. CD47 and its ligands, TSP1 and SIRPα, inhibit NK cell activation, proliferation, and function. The TSP1–CD47 axis activates the JAK–STAT3 signalling pathway in NK cells and blocking STAT3 can restore IFN-γ production by NK cells, indicating that CD47 and its related signalling pathways could be targets for improving NK cell function in people living with HIV (Fig. 6C).

## Notes

The experimental reagents or resources are shown in Additional file 1: Table S2.

### Supplementary Information

**Additional file 1: Figure S1.** TSP1–CD47 axis inhibits NK cells activation, proliferation, and IFN-γ production. **A** Schematic diagram of experiments and representative cytometry dot plots using NK cells from NC group treated with different concentrations of recombinant TSP1 (0, 250, 500, and 1000 ng/mL) to study TSP1–CD47 axis effects on proliferation (Ki-67 expression) and activation (CD69 expression) of human NK cells. **B** Schematic diagram of experiments and representative cytometry dot plots using NK cells from NC group treated with recombinant TSP1 (1000 ng/mL) or Anti-CD47 antibody (2 μg/mL) to study TSP1–CD47 axis effects on IFN-γ production of human NK cells. **C** Paired comparisons of the percentage of IFN-γ expression in total NK cells with recombinant TSP1 (1000 ng/mL) treatment and anti-CD47 antibody (2 μg/mL) or IgG-control (2 μg/mL) treatment (n = 7). Paired-t or Wilcoxon signed-rank test was used to make paired-group comparisons, and error bars represent median and interquartile range; *p < 0.05, **p < 0.01, ***p < 0.001; ns, no significance; NT, no treatment. **Figure S2.** Blocking SIRPα restores IFN-γ production of NK cells via NFAT signaling pathway in PLWH. **A** Schematic diagram of experiments and representative cytometry dot plots using NK cells from people living with HIV treated with anti-SIRPα antibody (2 μg/mL) or IgG-control (2 μg/mL) to study effects on IFN-γ production of human NK cells. **B** Paired comparisons of the percentage of IFN-γ expression in total NK cells from the people living with HIV with anti-SIRPα antibody (2 μg/mL) or IgG-control (2 μg/mL) treatment (n = 7). **C** Schematic diagram of experiments and representative cytometry dot plots using NK cells from people living with HIV treated with Anti-SIRPα antibody (2 μg/mL) or IgG-control (2 μg/mL) to study effects on the phosphorylation of the nuclear factor of activated T-cells (NFAT) signaling pathway in human NK cells. Blue, Anti-SIRPα antibody treatment; red, IgG-control treatment. **D** Paired comparisons of the percentage of NFAT phosphorylation in total NK cells from people living with HIV with anti-SIRPα antibody (2 μg/mL) or IgG-control (2 μg/mL) treatment (n = 3). A paired t-test was used for paired-group comparisons, and error bars represent the median and interquartile range; **p < 0.01, ***p < 0.001. **Figure S3.** TSP1–CD47 axis activates STAT3 signal pathway of NK cells. **A** Schematic diagram of experiments and representative cytometry dot plots using NK cells from NC group treated with or without recombinant TSP1 (1000 ng/mL) to study TSP1–CD47 axis effects on phosphorylation of STAT3 signaling pathway of human NK cells. **B** Schematic diagram of experiments and representative cytometry dot plots using NK cells from people living with HIV treated with recombinant TSP1 (1000 ng/mL) and with or without STAT3-inhibitor Stattic (2 µmol/L) to study STAT3 blockade effects on IFN-γ production of human NK cells. **Table S1.** Characteristics of subjects enrolled in this study. **Table S2.** Key resources.

## Data Availability

All of the relevant data and materials are available from the authors upon reasonable request.

## Abbreviations

HAART: Highly active antiretroviral therapy
CD47: Cluster of differentiation 47
GO: Gene ontology
GSEA: Gene set enrichment analysis
HIV: Human immunodeficiency virus
IFN-γ: Interferon-gamma
IL: Interleukin
JAK: Janus Kinase
NFAT: Nuclear factor of activated T cells
NK: Natural killer cell
PBMCs: Peripheral blood mononuclear cells
PD-1: Programmed cell death protein 1
PRRs: Pattern recognition receptors
RNA-seq: RNA sequencing
SIRPα: Signal regulatory protein α
ssRNA: Single-stranded ribonucleic acid
STAT3: Signal transducer and activator of transcription 3
TSP1: Thrombospondin-1
TLR: Toll-like receptor
