# Supplementary material for: Inhibitory receptor CD47 binding to plasma TSP1 suppresses NK-cell IFN-γ production via activating the JAK/STAT3 pathway during HIV infection
Source: J Transl Med. 2023 Nov 30;21:869. doi: 10.1186/s12967-023-04667-6 (PMC10688093; doi:10.1186/s12967-023-04667-6)
Supplement: Supplementary file 1 — Additional file 1: Figure S1. TSP1–CD47 axis inhibits NK cells activation, proliferation, and IFN-γ production. A Schematic diagram of experiments and representative cytometry dot plots using NK cells from NC group treated with different concentrations of recombinant TSP1 (0, 250, 500, and 1000 ng/mL) to study TSP1–CD47 axis effects on proliferation (Ki-67 expression) and activation (CD69 expression) of human NK cells. B Schematic diagram of experiments and representative cytometry dot plots using NK cells from NC group treated with recombinant TSP1 (1000 ng/mL) or Anti-CD47 antibody (2 μg/mL) to study TSP1–CD47 axis effects on IFN-γ production of human NK cells. C Paired comparisons of the percentage of IFN-γ expression in total NK cells with recombinant TSP1 (1000 ng/mL) treatment and anti-CD47 antibody (2 μg/mL) or IgG-control (2 μg/mL) treatment (n = 7). Paired-t or Wilcoxon signed-rank test was used to make paired-group comparisons, and error bars represent median and interquartile range; *p < 0.05, **p < 0.01, ***p < 0.001; ns, no significance; NT, no treatment. Figure S2. Blocking SIRPα restores IFN-γ production of NK cells via NFAT signaling pathway in PLWH. A Schematic diagram of experiments and representative cytometry dot plots using NK cells from people living with HIV treated with anti-SIRPα antibody (2 μg/mL) or IgG-control (2 μg/mL) to study effects on IFN-γ production of human NK cells. B Paired comparisons of the percentage of IFN-γ expression in total NK cells from the people living with HIV with anti-SIRPα antibody (2 μg/mL) or IgG-control (2 μg/mL) treatment (n = 7). C Schematic diagram of experiments and representative cytometry dot plots using NK cells from people living with HIV treated with Anti-SIRPα antibody (2 μg/mL) or IgG-control (2 μg/mL) to study effects on the phosphorylation of the nuclear factor of activated T-cells (NFAT) signaling pathway in human NK cells. Blue, Anti-SIRPα antibody treatment; red, IgG-control treatment. D Paired [file 12967_2023_4667_MOESM1_ESM.docx]

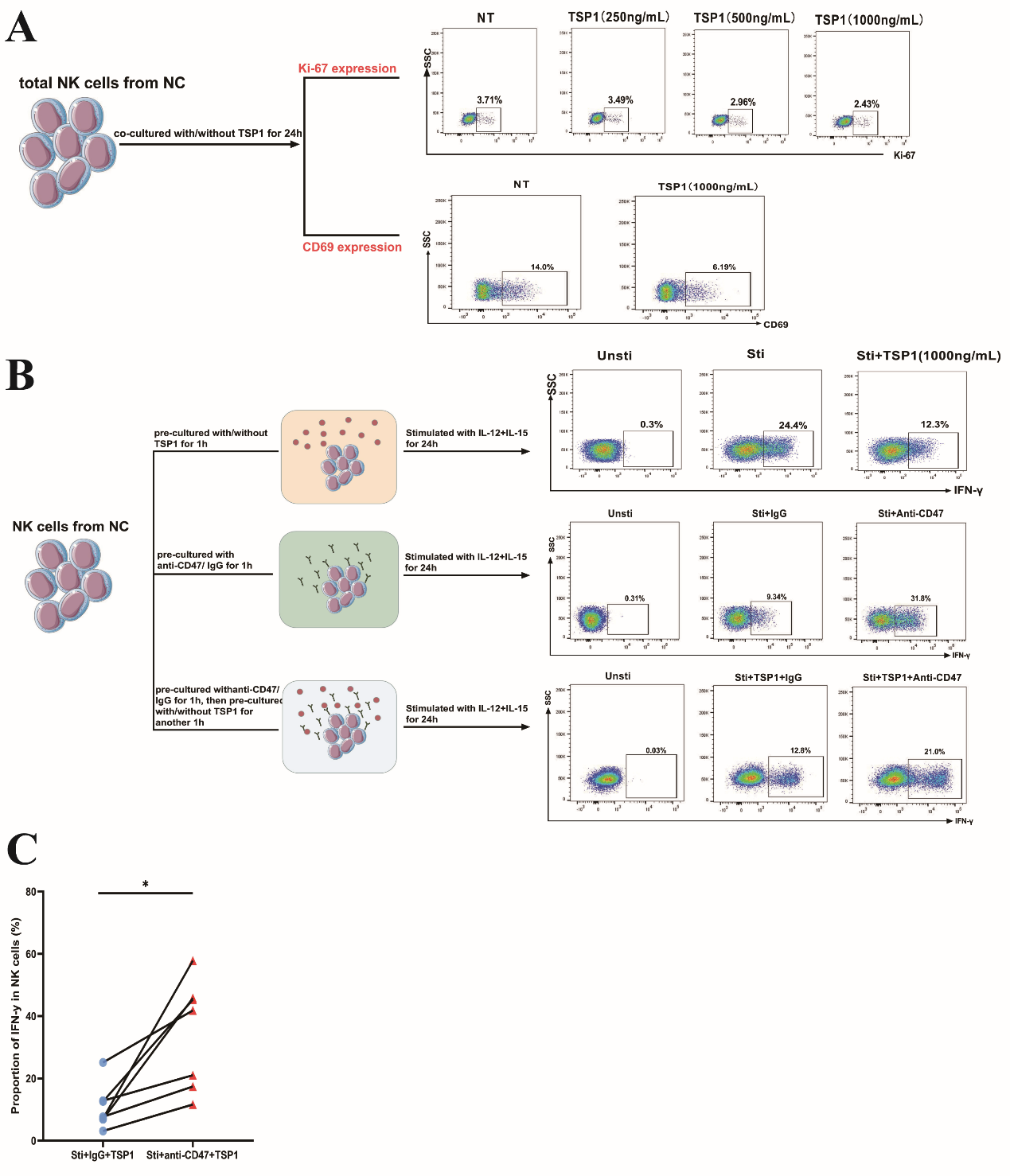


**Figure S1** **TSP1–CD47 axis inhibits NK cells activation, proliferation, and** **IFN-γ production** **(A)** Schematic diagram of experiments and representative cytometry dot plots using NK cells from NC group treated with different concentrations of recombinant TSP1 (0, 250, 500, and 1000 ng/mL) to study TSP1–CD47 axis effects on proliferation (Ki-67 expression) and activation (CD69 expression) of human NK cells. **(B)** Schematic diagram of experiments and representative cytometry dot plots using NK cells from NC group treated with recombinant TSP1 (1000 ng/mL) or Anti-CD47 antibody (2 μg/mL) to study TSP1–CD47 axis effects on IFN-γ production of human NK cells. **(C)** Paired comparisons of the percentage of IFN-γ expression in total NK cells with recombinant TSP1 (1000 ng/mL) treatment and anti-CD47 antibody (2 μg/mL) or IgG-control (2 μg/mL) treatment (n = 7). Paired-t or Wilcoxon signed-rank test was used to make paired-group comparisons, and error bars represent median and interquartile range; ^*^*p* < 0.05, ^**^*p* < 0.01, ^***^*p* < 0.001; ns, no significance; NT, no treatment.


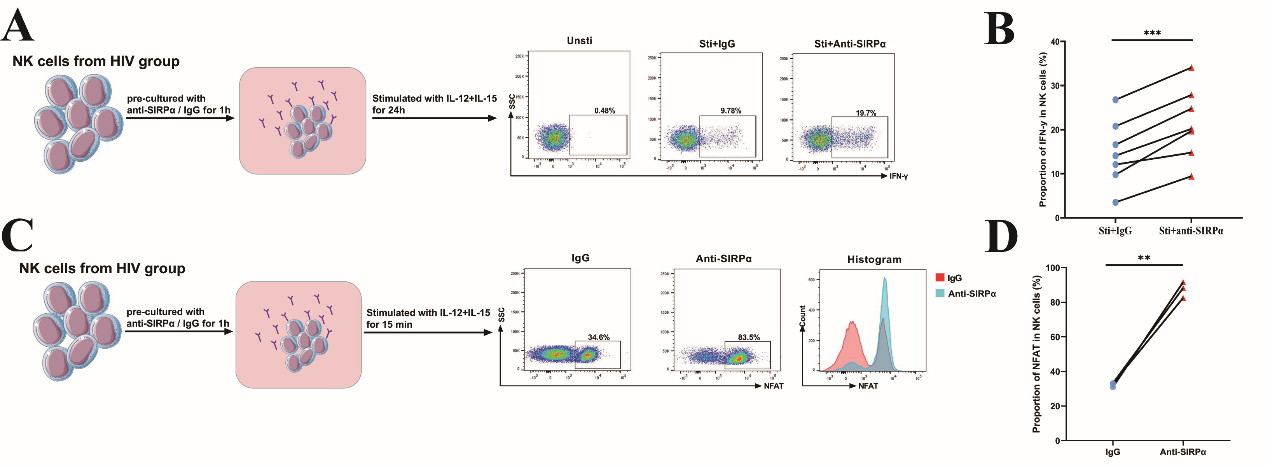


**Figure S2** **Blocking SIRPα restores IFN-γ production of NK cells via NFAT signaling pathway in PLWH (A)** Schematic diagram of experiments and representative cytometry dot plots using NK cells from people living with HIV treated with anti-SIRPα antibody (2 μg/mL) or IgG-control (2 μg/mL) to study effects on IFN-γ production of human NK cells. **(B)** Paired comparisons of the percentage of IFN-γ expression in total NK cells from the people living with HIV with anti-SIRPα antibody (2 μg/mL) or IgG-control (2 μg/mL) treatment (n = 7). **(C)** Schematic diagram of experiments and representative cytometry dot plots using NK cells from people living with HIV treated with Anti-SIRPα antibody (2 μg/mL) or IgG-control (2 μg/mL) to study effects on the phosphorylation of the nuclear factor of activated T-cells (NFAT) signaling pathway in human NK cells. Blue, Anti-SIRPα antibody treatment; red, IgG-control treatment. **(D)** Paired comparisons of the percentage of NFAT phosphorylation in total NK cells from people living with HIV with anti-SIRPα antibody (2 μg/mL) or IgG-control (2 μg/mL) treatment (n = 3). A paired t-test was used for paired-group comparisons, and error bars represent the median and interquartile range; ^**^*p* < 0.01, ^***^*p* < 0.001.


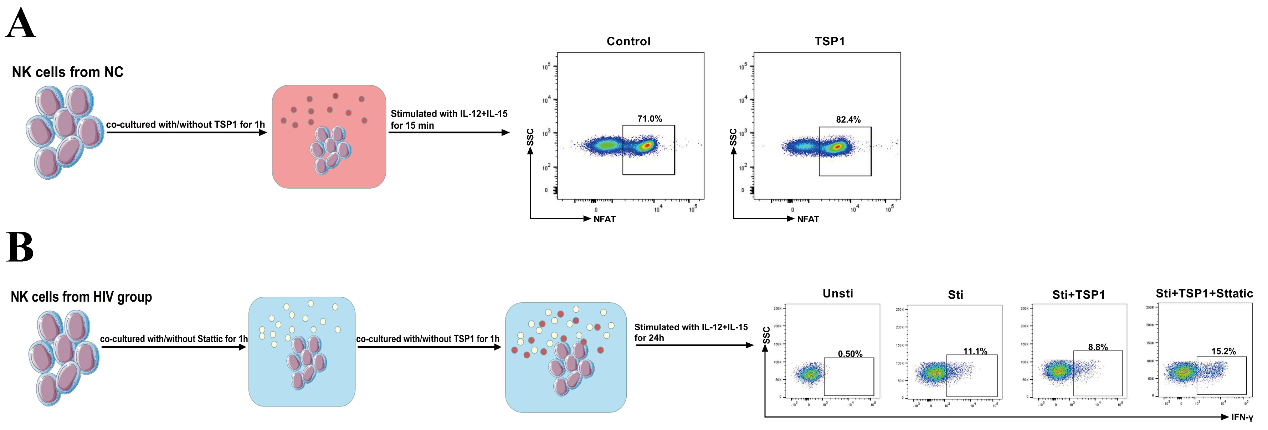


**Figure S3** **TSP1-CD47 axis activates STAT3 signal pathway of NK cells (A)** Schematic diagram of experiments and representative cytometry dot plots using NK cells from NC group treated with or without recombinant TSP1 (1000 ng/mL) to study TSP1–CD47 axis effects on phosphorylation of STAT3 signaling pathway of human NK cells. **(B)** Schematic diagram of experiments and representative cytometry dot plots using NK cells from people living with HIV treated with recombinant TSP1 (1000 ng/mL) and with or without STAT3-inhibitor Stattic (2 µmol/L) to study STAT3 blockade effects on IFN-γ production of human NK cells.
